# A Trial of a Virtual Fence to Mitigate Roadkill on an Unsealed Rural Road in Tasmania, Australia

**DOI:** 10.3390/ani14111641

**Published:** 2024-05-31

**Authors:** Steven G. Candy, James A. Bunker, Bruce Englefield

**Affiliations:** 1 Scandy Statistical Modelling Pty Ltd., 70 Burwood Drive, Blackmans Bay, TAS 7052, Australia; 2 Independent Researcher, Lunawanna, South Bruny Island, TAS 7150, Australia; 3 Independent Researcher, Lindisfarne, TAS 7015, Australia

**Keywords:** wildlife vehicle collisions, roadkill, one welfare, virtual fence, avoidance learning, animal welfare

## Abstract

**Simple Summary:**

Simple Summary: Roadkill of native animals, particularly marsupials, is a serious problem for wildlife conservation, animal welfare, and tourism in Tasmania, so any proposed method that substantially reduces native marsupial roadkill, while being cost-effective, deserves a thorough scientific evaluation. A commercial roadkill Virtual Fence (VF) mitigation device was evaluated using a repeat of a similar trial in southern Tasmania in 2018. The same VF installation and experimental designs were employed over a similar length of road (4.9 km versus 4.5 km), with the current trial having a substantially longer period of monitoring (i.e., 585 d versus 126 d). Sections of the virtual fence were switched on or off according to the same predetermined experimental design. Tasmanian pademelons were the most commonly killed species, at 222, over the total trial period, while 47 Bennett’s wallabies were killed. The kill rate for the three spatial replicates and two monitoring periods (a total of 110 pademelons killed over 221 d) when the VF was switched off versus the periods when it was switched on were 3.35 and 3.33 month^−1^km^−1^, respectively. The corresponding rates for the total of 19 Bennett’s wallabies that were killed were 0.55 and 0.61. Analyses of pademelons, which included pre-on and post-on periods for comparison, failed, as in the 2018 trial, to demonstrate any statistically significant effect of the virtual fence in reducing roadkill for this species.

**Abstract:**

A commercial roadkill Virtual Fence (VF) mitigation device (iPTE Traffic Solutions) was used in a field trial to test its effectiveness, for which previously published results have been inconsistent, along a 4.9 km segment of road on Bruny Island, Tasmania. A total of 585 days of monitoring roadkill by species was conducted, with six sections that were alternatively switched on or off according to the Crossover and Multiple Before–After–Control–Impact (MBACI) experimental designs that divided monitoring into “off–on” then “on–off” periods. Aggregate counts, for each period by section combination, from daily counts of Tasmanian pademelons (*Thylogale billardierii*) were modelled, with a total count of 222. The statistical analysis used the MBACI design to estimate the VF effect using a log-odds ratio parameter (LORP) while accounting for local spatio-temporal effects. Both versions of the analysis, either averaged over the three spatial replicates (paired sections) or two temporal replicates (blocks), showed no statistically significant effect of the VF, judged as an LORP estimate not sufficiently below zero. Corresponding percentage reduction estimates of 9% and 16% were derived from the LORP. The corresponding statistical power required to detect a nominal significant reduction of 50% in rate was 0.5 and 0.6, respectively. This study confirms the results from a similar previous field trial in southern Tasmania that this VF is likely to lead to, if anything, only a minor reduction in roadkill.

## 1. Introduction

Wildlife vehicle collisions (WVC), producing what is colloquially know as roadkill, can have serious consequences for animals and humans, i.e., death or injury, and can affect the environment through species decline [1,2,3,4]. Three main approaches to mitigating the problem of WVC, which can be undertaken individually or in combination, are infrastructure management, changing human behaviour, and changing animal behaviour [5,6,7,8,9,10]. We describe the results of a field trial in southern Tasmania to evaluate the effectiveness of an implementation of the last-mentioned approach [10] using a solar-powered, transport system sensor/actuator manufactured in Austria (iPTE Traffic Solutions Ltd., 8054 Graz/Austria Mantscha-Wald-Weg 48, Austria). The units are designed to produce a virtual fence (VF) along the roadway and to work from dusk to dawn, with the units being sequentially triggered by oncoming vehicle headlights. The operation of the units is meant to alert crepuscular and nocturnal animals [10]. A three-year trial in Tasmania of this VF, starting in 2014 [11], estimated that roadkill of Tasmanian pademelons (*Thylogale billardierii*) was reduced by over 50% and that “these devices have enormous potential to substantially reduce roadkill rates” [11]. A serious weakness in that study was that the main comparison relied on temporal pseudo-replication [12] (i.e., monthly counts over which there was no change in the allocation of treatments of “fenced” and “unfenced” road sections) for statistical tests (paired t-tests involving the “fenced/unfenced” contrast), and which led to a reduction rate close to 50% reported for pademelons. That study design employed no spatial replication of the control (unfenced) and intervention (fenced) road sections, thus introducing the possibility of the intervention effect being confounded with the road section effect [12]. Coulson and Bender [13] noted other criticisms of that study to which the authors responded [14]. In contrast, the 2018 trial described by [15] used the “interspersed treatments” requirement for “good experimental design”, as recommended by [12], by employing three spatial replicates with two temporal replicates in a replicated or multiple Before–After–Control–Impact design (MBACI) [16]. The results from this trial did not show any statistically significant reduction in roadkill rate for the three predominantly killed native marsupial species: Tasmanian pademelons (TP), Bennett’s wallabies (*Notamacropus rufogriseus*) (BW), and common brushtail possums (*Trichosurus vulpecula*). However, as acknowledged by [15], a limitation of that study was the relatively short overall monitoring period of 126 d, including a total of 56 d when the VF was switched on, resulting in relatively small number of roadkill and thus reducing the precision of the estimate of the effect of the VF [15]. Despite this, the statistical power required to detect an effect size of 50% reduction was found by simulation to be adequate due to the replication described above. Further, a potential reduction in the effectiveness of the VF due to site-specific effects, particularly the high speed of the highway traffic for the 2018 trial, was acknowledged by [15]. The equipment manufacturers state that the ‘effectiveness [of the VF devices] is speed dependent’. Also, the volume of sound surrounding the road may also impact its effectiveness, as demonstrated by Englefield et al. [15].

The trial reported here is a repeat of the 2018 trial [15], also carried out in southern Tasmania, Australia, and starting in April 2020, which used the same VF installation, field methods, and experimental trial design over a similar length of road (4.9 km versus 4.5 km in [15]); however, the trial described here was not limited to a short duration, having substantially longer periods of monitoring both overall and when the VF was switched on. We rely on the descriptions in [15], concentrating on describing any differences between the two trials. Importantly, we aimed to assess the operation of the VF in a spatially different environment from the previous trials conducted in Tasmania. For these reasons, a site on Bruny Island, Tasmania, was chosen, where the speed limit is 80 km/h and the traffic volume is unlikely to cause a sufficient volume of sound to interfere with the VF operating conditions. In addition, rather than applying the four different and complementary statistical analysis methods used by [15], we used a modified version of one of those methods, as described by [17], which exploits the MBACI experimental design feature and models the counts using discrete distributions of Poisson or a negative binomial if the former was not adequate in explaining any overdispersion of the counts.

Therefore, the objective of this study was to obtain another site-specific estimate of the effect of the VF, while improving the precision of the estimate compared to [15] by substantially increasing the number of days that were monitored overall and the number of days on which the VF was switched on.

## 2. Materials and Methods

### 2.1. Study Site

Study site: In December 2020, Kingborough Council erected the VF system of IPTE Traffic Solutions Ltd. (Graz, Austria) on a section of Cloudy Bay Road, South Bruny Island, Tasmania. Cloudy Bay Road is 9.9 km and starts at a T-junction driving south on Bruny Island’s main road. Cloudy Bay road only goes to Cloudy Bay National Park and the Great Southern Ocean. The study area was 5.5 km of Cloudy Bay Road, finishing near the end of the road at East Cloudy Bay National Park. The GPS co-ordinates for the start of the VF fence are 43°23′51″ S and 147°15′14″ E. The GPS co-ordinates for the finish are 43°26′21″ S and 147°14′38″ E (Figure S1, Supplementary Material), providing a length of 4.9 km. The study area was chosen as it was identified as a roadkill hotspot on Bruny Island by a preliminary whole Island Citizen Science project of the Bruny Island Environment Network and Dr Bruce Englefield, University of Sydney. Roadkill was monitored and reported through the use of a mobile phone application: “Roadkill Reporter”. There are few permanent residents. The high frequency and intensity of the traffic volume are a function of both national and international tourism.

Road Description: Cloudy Bay Road is a two-way unsealed road with a speed limit of 80 km/h. It is mainly straight with some tight bends. It is gently undulating with no hills. The study road section has gentle bends, with a slightly tighter bend at the southern extent (Figure S1, Supplementary Material). The road travels through cleared paddocks, sometimes on both sides, and drained natural wetlands. Eucalypt forest reaches sections of the road on the eastern side, and these narrow strips between the cleared areas extend from the South Bruny National Park (Figure S1 Supplementary Material). This provides a large wooded area and a habitat and refuge for the animals of Bruny Island; some of these are represented in the roadkill. There are some hobby farms with small herds of sheep *(Ovis aries*) and alpacas (*Lama pacos*). The study area included in situ fencing ranging, in sections, from wallaby-proof fencing to loose, three-strand, wires on posts and no fencing at all. There were no streetlights in the study area.

### 2.2. Subject Species

From the Citizen Science project, it was known that the predominant species that was expected to be killed in the trial would be Tasmanian pademelon and, less commonly, Bennett’s wallaby. Tasmanian pademelons are small marsupials in the genus *Thylogale*, also known as the rufous-bellied pademelon, and are the sole species of pademelon found in Tasmania. *T. billardierii* is the largest species in the genus (e.g., mean body weight of males–females is 9.0:5.8 kg) [18]. Their brown fur and lack of facial markings make them difficult to see at night in the headlights of a car and they exhibit little eye shine. Their maximum speed of travel is 50 km/h. Bennett’s wallabies, also called red-necked wallabies, can weigh between 14 and 19 kg and attain a head–body length of 900 mm, with males generally being bigger than females [19].

### 2.3. Sampling Methods, Experimental Design, and Data Collection

The sampling methods and experimental design followed that of [15], with a few exceptions. The VF was installed on six road sections, each of approximately 750 m length, with a short 450 m section between the 2nd and 3rd installed sections that did not have the VF installed, but which was monitored, along with the above sections, for the duration of the trial (see Figure S1, Supplementary Material). GPS and Google maps were employed to measure and allocate the exact GPS co-ordinates of the start and end of each section.

After numbering the above sections as 1 to 7 from north to south, the sections were paired, starting from the north with the VF-free Section 3 incorporated into pair 1 (i.e., paired through coding but included separately in the dataset of aggregated counts). This led to three pairs, denoted as spatial replicates (Reps 1, 2, 3). Roadkill monitoring began on 2 April 2020 and monitoring “periods” corresponded to the set of consecutive days on which the VF was either not yet installed (Period 1, 136 d) or subsequently installed but switched “off” or “on” for set periods. There were two blocks of periods in which the BACI design was repeated, but the section within a pair for which the VF was switched on was reversed. This reversal led to a cross-over design [20]. Period 2 (70 d) started when the VF was installed on 8 December 2020 but switched off for this period. For Period 3 (120 d), the northerly section in each pair was switched on (Block 1); the next period, Period 4 (16 d), was a “wash-out” period when all VF posts were switched off. This was followed by Period 5 (101 d) with the southerly section (i.e., the VF-installed Section 2 in Rep 1) in each pair switched on. This was followed by the nominated second wash-out 16 d period (Period 6); the VF was switched off for all sections at the start of this period. For the final period of monitoring, Period 7 (126 d), the VF remained switched off for all sections. The wash-out periods were excluded from analyses, as is standard for cross-over designs. This gave three spatial by two temporal replicates of the BACI design, noting that the “Impact” (i.e., VF on) was applied in the “Before” period and the “Control” (i.e., VF off/uninstalled) was applied in the “After” period in Block 2 as the “on–off” temporal combination, reversing the order to that of Block 1. This is unusual for BACI designs because, in most applications, the “Impact” (e.g., fixed infrastructure) cannot simply be turned off (or removed) and on (or installed) at will as the VF can. However, the validity of the standard BACI analysis (see [17]) was maintained by applying appropriate coding of the sections for each periods with respect to the BACI categories (see Table S1 in the Supplementary Material). Note that, in [15], there was no extended period of monitoring after the VF was switched off for Block 2, so the period prior to switching on the VF for Block 2 was used as the “Control” period, as in the standard definition of a BACI design.

The study area was monitored daily, and sampling was undertaken every day except when weather conditions or time constraints interfered. Consecutive day monitoring failed to occur on only four occasions. Malfunctioning units were immediately replaced. Dead and injured animals were counted and photographed (and euthanised where necessary when there was no prospect of rehabilitation); the roadkill animal and the GPS co-ordinates obtained from a separate hand-held Garmin GPS 60, as well as the photographs, GPS data, and species identification, were then entered into a spreadsheet. The carcasses were removed from the road, which was clear of overhead power lines and fencing. This was to ensure that no double-counting of roadkill occurred and also to reduce the likelihood of secondary roadkill due to scavenging by a wide range of raptors and Eastern quoll, (*Dasyurus viverrinus*).

Daily counts, given the above caveat, of roadkill for each species were aggregated by period, with Periods 1 and 2 kept separate and coded as “Before” and “Control”, excluding wash-out periods. This led to a dataset that could be for the analysis for each species for seven sections over five periods. This gave 35 counts, corresponding to seven sections, divided into contiguous pairs of three Reps over five periods, with periods coded as belonging to one of two blocks according to the “off–on” VF status across Periods 1 to 3, with Period 3 being “on” and denoted as Block 1, and “on–off” for periods 5 and 7, denoted as Block 2. The counts of wash-out periods 4 and 6 were excluded from analyses. The dataset of the counts of TP roadkill and the description of the trial in terms of treatments, replicates, period lengths, and trial sections are given in table form in the Supplementary Materials (Table S1).

A traffic counter (Vehicle Classifier System, MetroCount, 15 O’Connor Close, North Coogee, W.A. 6163 Australia) was located at approximately 43°23′48″ S, 147°15′14″ E. The counter accurately approximates the number of vehicles using the full extent of the trial site and their speed on a continuous basis. This was in place for only 42 d (17 February 2021 until 31 March 2021) of the total trial period.

### 2.4. Statistical Analyses

#### 2.4.1. Simple Comparison of Rates

A simple, valid comparison of the kill rates between the fence when switched on versus off requires the use of counts from only two of the four periods in which the fence was switched on in three of the six VF-installed sections (i.e., including only the two “on” periods of the four “off–on” and “on–off” periods and excluding Section 3), where the total days monitored across Periods 3 and 5 was 221 d, whereas the equivalent in [15] was 56 d. These two selected periods thus provided a balanced dataset with respect to whether or not the VF was being operated, and allow for a simple rate comparison consisting of the total count for each “on” vs. “off” section, divided by the total across sections of the mathematical product of each section length and period length in units of km and month, respectively. This is equivalent to the weighted average of section rates where the weights are the product of the above. Note that this weighted average is scale-invariant with respect to length since, if the section length units were input as m rather than km, then this mean would be unchanged; this is similarly true for units of period length. The use of the counts for all monitoring periods and the specific advantages of the MBACI analysis in adjusting for the local spatial effects and temporal effects [17] described next justify the effort required for this more sophisticated analysis compared to the simple comparison presented above. However, the latter can be used as a common-sense check on the more complex analyses as their conclusions are similar.

#### 2.4.2. Fitting Poisson GLMs or Negative Binomial Extended GLMs to the MBACI Tables

We used the MBACI analysis of [17], implemented as a generalized linear model (GLM), for the count data with a logarithmic link function, including an “offset” of the Naperian logarithm of the product of the two exposure variables: the length of the section of road (km) and the length of the period (month, with nominal 30 d months). Candy and Englefield (2022) [17] analysed the dataset of counts of bare-nosed wombat roadkill in [21] using the above GLM. An assumed Poisson distribution for the counts was applied in [17] to the unreplicated BACI design of [21], which deployed the same VF used in this study. The statistical analysis that exploited the MBACI design to estimate the VF effect used the GLM to fit a log–odds ratio parameter (LORP) while accounting for local spatio-temporal effects. The LORP given by [17] is defined as the logarithm of the odds ratio of a random kill in the fenced section (s) that occurred during the post-installation (i.e., “on” VF status) period to the corresponding odds for the unfenced section (s) (i.e., “off” VF status). A null hypothesis of no effect of the VF corresponds to a log odds ratio of zero and the alternative hypothesis is that the LORP is sufficiently negative relative to parameter estimate uncertainty and the estimate’s assumed statistical distribution (the Gaussian distribution in [17]) to reject the null hypothesis. A point estimate and its uncertainty bounds for the size of the mitigation effect of the VF are also obtained as the LORP estimate. The LORP estimate can also be mathematically manipulated to provide a corresponding estimate of the percentage reduction in the rate of roadkill under the operation of the VF [17]. The LORP quantifies the effect of the VF using the “Before vs. After” comparison for the impact (i.e., VF “on” sections/periods), while adjusting for contemporaneous nuisance effects in the equivalent comparison for the control (i.e., VF “off” sections/periods) [17]. Thus, if the ratio of the rates (the After rates divided by the Before rates) for the VF “on” (i.e., the “Impact” in BACI) is, for example, one, and is combined with the corresponding but greater than one ratio for the VF “off” (i.e., the “Control” in BACI), then this gives a negative estimate for the LORP, suggesting a reduction in rate due to the VF if this is statistically significant from zero. Alternatively, if the above ratio for the VF “on” is less than one and the Control rate is one, then the LORP will also be negative. The first of these examples shows how the “control-adjustment” of the impact effect can be substantial. The relationship between these rate ratios and the LORP is given by [17]. Furthermore, the details of the specific GLM and how the LORP is defined within the GLM and thus estimated are given by [17]. The interpretation of the LORP, including how local spatio-temporal effects, such as those described above, are accounted for, is presented for the unreplicated BACI and its extension, based on simulation, to hypothetical spatial replication (i.e., MBACI trial) in [17]. Two versions of the analysis, either averaging the three spatial replicates (paired section or “Rep”) or two temporal replicates (“Block”), were carried out by fitting either of these factors as fixed effects and then averaging over each level while eliminating the between-level variance of effects to ensure greater precision in the average effect size. This was justified by the “detection device” average inference of [17] (preferred to inference for a random “across-population-units” estimated effect) for a first-stage “proof-of-concept” study. Fully implementing this approach leads to a single version of the model that calculates the VF effect by averaging across the six levels of the combination of Rep and Block factors. This involves fitting this interaction as a fixed effect. However, the fit of this model led to unstable and extreme estimates of some parameters, since some of these fixed effects were required to estimate zero observed counts. Therefore, it was necessary to consider Block versus Rep-averaging as separate implementations of this approach.

Due to the lack of replication in the single 2 × 2 BACI table modelled by [17], the Poisson assumption [22] could not be verified empirically. In this study, this assumption could be empirically examined for overdispersion relative to Poisson since there was a non-zero number of residual degrees of freedom for each model version. If there was evidence of significant overdispersion after comparing the residual deviance to its degrees of freedom (DF) (i.e., when this deviance was greater than the chi-square quantile corresponding to a 5% probability for the given DF, given the asymptotic distribution of the deviance [23]), a GLM with a negative binomial distribution for the counts was applied. The negative binomial (NB) is one way of accounting for overdispersion relative to a Poisson by positing that, rather than a fixed expected rate, for each given combination of predictor variables, the rate varies randomly according to an underlying gamma distribution [24].

The statistical significance of the estimated effect of the VF was judged by the estimated LORP’s to be a quantile of the null distribution. This suggested the corresponding probability of exceeding this quantile, as provided by the glm function in the R-software (Version 4.0.3) [25] when fitting a Poisson or the gam function of the mgcv library [26] when fitting a NB response distribution.

The NB fit in gam corresponds to an extension of the GLM fitting algorithm through the estimation of an extra variance parameter, θ, since the NB is not in the exponential family of distributions that standard GLMs are restricted to. The variance function for the NB adds an extra term to the simple one-to-one relationship with the expected value of the Poisson; this extra term is the square of the expected value divided by θ. The option of using the gam function in mgcv in the R-software to estimate θ by searching for a value that equates the Pearson chi-square statistic when divided by its degrees of freedom to a value of 1 was used here. The alternative approach to the NB GLM is to apply a quasi-Poisson response distribution [26] in the GLM to account for extra-Poisson dispersion. This approach retains the convenient properties of the Poisson but has less rigorous theoretical support than the NB.

Given the estimated uncertainty of the estimate of the LORP, the power of detecting an LORP corresponding to a 50% reduction as significant, assuming a Gaussian distribution for the estimate, was calculated as described by [17].

Alternative statistical analyses for the similar 2018 southern Tasmanian trial were used, including a similar MBACI analysis that used linear models for empirical rate as response variable and averaged the VF effect over the two temporal blocks [15]. All analyses results in that study were similar, so, to avoid a multiplicity of results, we restricted the analyses to the above glm and gam, parameterised according to the MBACI design.

## 3. Results

### 3.1. Data Summary and Simple Comparison of Rates

Tasmanian pademelons (TP) and Bennett’s wallabies (BW) accounted for most of the total roadkill of 295 native marsupials, with 234 TP killed over the total trial period and 12 killed in the wash-out periods, while 49 BW were killed, with 2 killed in the wash-out periods. Ten common brushtail possums (*Trichosurus vulpecula*) were killed.

**Figure 1:**
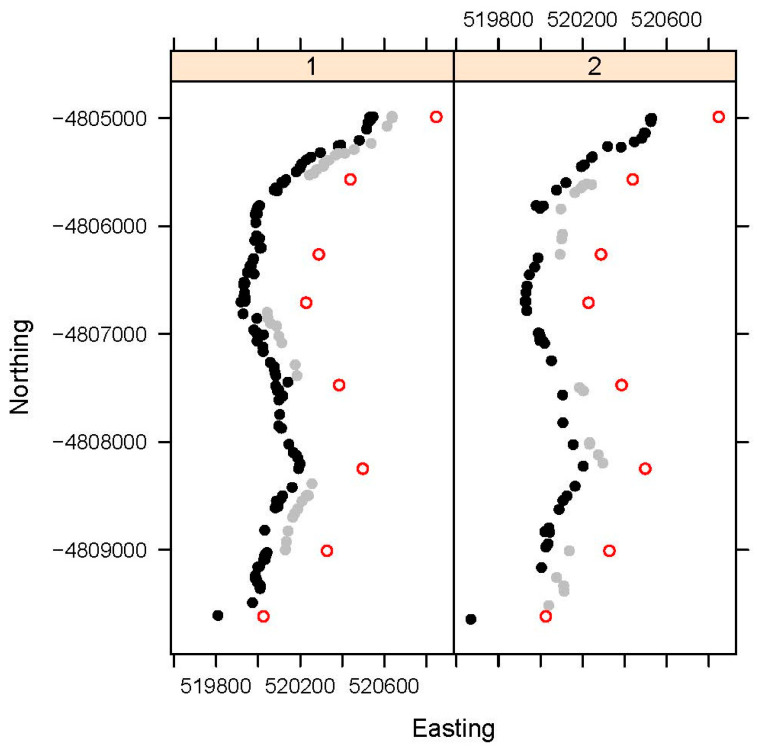
Locations of Tasmanian pademelon (*Thylogale billardierii*) roadkill for Block 1 (Periods 1, 2, and 3) and Block 2 (Periods 5 and 7), showing the locations of the section start/end posts, artificially offset by 300 on the Easting scale for clarity and denoted in the text as Posts 1 to 8 (red open circles), starting from the northern end of the road section. Roadkill that occurred when the VF was switched off are shown as black filled circles, and roadkill that occurred when VF was switched on are shown as grey filled circles, with the latter offset by 100 on the Easting scale for clarity. VF posts were alternately located every 25 m along the road (i.e., 50 m between posts on the same side) and no VF posts were installed in the short Section 3 between section start/end posts 3 and 4. All sections, when switched either on or off, recorded some roadkill in Blocks 1 and 2.

Spatial locations for TP roadkill are given in Figure 1.

For the three replicate on–off pairs of road sections (excluding Section 3) for each of the two “on” monitoring periods (i.e., only one section in each pair was switched on) of Periods 3 and 5, a total of 110 TP were recorded to be killed over the 221 d. For the three sections of each of the two blocks when the VF was switched off, a simple comparison of the TP kill rate when those sections were switched on gave weighted average rates of 3.35 versus 3.33 month^−1^km^−1^, respectively. The corresponding rates for the total of 19 BW were 0.55 and 0.61.

The median speed was obtained from the traffic counter using 9971 vehicle passes over the 42 d period in which it was deployed and included both daytime and dusk-to-dawn passes. The median speed was 60.1 km/h, with 5.6% of recordings being greater than the 80 km/h speed limit.

### 3.2. Fitting Poisson GLMs or Negative Binomial Extended GLMs to the MBACI Tables

Both versions of the analysis, either averaging the three spatial replicates (each of the paired sections or “Rep”) or two temporal replicates (“Block”), gave no statistically significant effect (*p* > 0.05) of the VF, judged as an LORP estimate not sufficiently below zero (Table 1) relative to a critical value of the appropriate Student’s t distribution with the probability level provided by gam. Corresponding percentage reduction estimates of 9% and 16% were derived from the LORP estimates (Table 1). Applying the parameter uncertainty estimates for the overall average LORP parameter, the power to detect an LORP corresponding to a 50% reduction in rate as significant, assuming a Gaussian distribution for “Rep” and “Block” fixed-effects averaging, was 0.5 and 0.6, respectively. The corresponding table to Table 1 for the quasi-Poisson GLM is given as Table S2 in the Supplementary Material.

## 4. Discussion

The overall effect of the VF on rates was quantified by both the LORP estimate and its conversion to a percentage reduction in rate estimate, as given in Table 1 in the “Average LORP” and “Percentage rate reduction” columns, respectively. Both versions of the analysis, either averaging the three spatial replicates (paired sections) or the two temporal replicates (blocks), showed no statistically significant effect of the VF, judged as an LORP estimate not sufficiently below zero. Interestingly, the spatial “Rep”-specific estimates of LORP both gave positive northerly and southerly Rep estimates for the triplicate, indicating an actual increase in rate when the VF was switched on, while the middle Rep gave a substantially larger (in absolute value) negative estimate, with corresponding estimates of percentage reductions of −38.5%, −98.2%, and 72.8%, respectively. None of the above estimates of LORP gave rise to a rejection of the null hypothesis of a zero (i.e., no effect) value (Table 1), as was the case for the overall average estimate of LORP. Evenso, this is a large range in estimates, which is especially noticeable when expressed as percentage reductions. This provides a clear warning that unreplicated BACI designs [21] or simpler, spatially unreplicated Control vs. Fenced designs [11] can confound nuisance spatial effects with the inferred effect of the “Impact” (i.e., operating VF) relative to the “Control”, since the middle spatial replicate in this trial would, on its own, provide a rather positive view of the benefit of the VF.

Further, despite the lack of statistical significance for the middle spatial replicate it would be tempting to consider that the associated uncertainty would be removed by further periods of monitoring or repeats of the single-replicate design combined with meta-analyses. Given this, the attraction of a practically useful 70% reduction in roadkill rate could result in a less cautious approach than that contingent on results from additional trials. Alternatively, the use of pseudo-replication could result in a statistically significant reduction due to the under-estimation of effect estimate uncertainty, since arbitrary divisions for long monitoring periods obtained using, for example, monthly counts can deliver large numbers of temporal pseudo-replicates (e.g., 38 months in [11]), compared to the much smaller numbers of true spatial replicates (three here) and temporal replicates (two here). However, using all three spatial replicates within what is a very uniform site with respect to the road properties, surrounding environment, and traffic behaviour leads to very different results. These results suggest a highly noisy random process of WVCs, expressed here as observed roadkill, combined with no substantial signal from the mitigation device that was deployed, resulting in an inconsistent “effect” that is either wildly positive or negative, as displayed by the percentage reduction estimates for spatial replicates in this study.

The same arguments apply to true versus pseudo-replications at the temporal scale since, in this study and in [15], each temporal “Block” replicate involves a different (i.e., reverse) allocation of treatment (i.e., VF “on” or “off”) to the pairs of road sections within Rep, whereas the months, which serve as pseudo-replicates in [11], had the same treatment allocation within each road section. As can be seen in Table 1 and in Figure 2 of [15], the block-level estimates of the effect of the VF are also highly inconsistent.

The spatial and temporal variability in the LORP presented above explains the lack of a statistically detectable effect of the VF, given that the statistical power of the design (improved with longer monitoring periods and with selection of “hotspot” sites) is adequate to detect a practically significant reduction in rate, which was, in this case, set at 50%. Low statistical power can also result in a potentially modest sized effect being missed, as reflected in the close-to-zero point estimate of the LORP [17]. The statistical power required to detect a nominal 50% reduction in rate, estimated for this study using the method of [17], was not as high, with a range of 0.5 to 0.6 compared to the comparable three-spatial-replicate simulation study by [17], that gave a power of 0.7. However, the simulation study used a theoretical Poisson distribution with no over-dispersion, whereas, with the real-world data, the over-dispersion was statistically significant (Table 1) and substantial, requiring an alternative discrete distribution that accounts for such over-dispersion. To achieve this, we applied the negative binomial. The alternative use of the quasi-Poisson GLM (Supplementary Material) gave similar results to the negative binomial GLM in terms of statistical significance but gave a slightly higher estimate of percent reduction by approximately an extra 5%.

In terms of the traffic speed for this site, it would seem that a lower speed limit of 80 km/h, compared to the 100 km/h highway speed limit for the other trial site in southern Tasmania [15], did not improve the efficacy of the VF, possibly because it was still above the maximum speed recommended by [27].

The sole objective of this study was to empirically test whether the VF has any effect on reducing roadkill of a native marsupial species for which the roadkill numbers are sufficient to obtain a good chance of detecting a practically useful reduction. Given this objective, our technique was to “design out” the background spatial and temporal effects of the exposed animal population and traffic conditions over space (habitat effects) and time (season effects) using replication and restricted randomisation within an appropriate experimental design; a technique pioneered at Rothamsted Research Station, UK, in the early 1900s for agricultural field trials, which has been a standard scientific method since then [28]. We used a combination of replicated BACI and cross-over designs to ensure that the Control versus Treatment (i.e., VF “On”) comparison was valid and its quantitative estimate via the LORP was as informative and precise as possible given the available resources. This study was never designed to be a study of the ecological drivers of background roadkill prevalence for this species of Tasmanian pademelon, or to be generalisable to other marsupial species or the complete range of ecologically diverse sites. However, it does provide additional evidence to that of [15], which also showed a minimal, if any, benefit of this mitigation device.

## 5. Conclusions

This study confirms the results of a similar field trial carried out in 2018, also in southern Tasmania, described by [15], and adds to the evidence that this VF is likely to lead to, if anything, only a minor reduction in roadkill for a commonly killed species for which researchers have the best chance of detecting any practically useful reduction. Using simple comparisons that restricted the data to balanced numbers of sections with the VF switched “on” versus “off”, with equivalent periods being monitored in each state, a comparison of rates indicated that there was no reduction due to the VF operation for both TP and BW; in fact, a slight increase was observed for BW. The above conclusion for TP was confirmed by the more sophisticated MBACI/gam analyses, which used all the data and provided estimates of the percentage reduction (or possibly inflation) of rates based on LORP estimates, along with the statistical uncertainty estimates for these estimates. Statistical hypothesis tests with a null hypothesis of no effect quantified by a zero LORP, or, equivalently, a zero percentage reduction in rate, versus the alternative hypothesis of a reduction in rate, were not rejected. The statistical power to detect a nominal 50% reduction in roadkill rate for Tasmanian pademelons using the MBACI design combined with the variability in roadkill section/period counts was reasonable, but could be improved in similar studies using more spatial and temporal replicates.

The data and R-code used in this study are provided in the Supplementary Material.

## Figures and Tables

**Table 1:** Results of the NB extended GLM fit to MBACI trial Tasmanian pademelon roadkill counts, quantifying the effect of the VF using LORP estimates.

Fixed Effect	LORP Estimates (SE, *t*-Value)			Average LORP (SE, *t*-Value)	Percentage Rate Reduction	NB θ Estimate (Poisson Deviance, DF)
	1	2	3			
Rep	0.326 (0.655, 0.50 ^ns^)	−1.301 (0.804, −1.62 ^ns^)	0.684 (0.807, 0.85 ^ns^)	−0.097 (0.438, −0.22 ^ns^)	9.2	4.8 (52.5 **, 23)
Block	0.254 (0.433, 0.59 ^ns^)	−0.600 (0.620, −0.97 ^ns^)		−0.172 (0.378, −0.46 ^ns^)	15.9	11.6 (47.7 *, 27)

^ns^ *p* > 0.05, * *p* < 0.01, ** *p* < 0.001 (one-sided tests).

## Data Availability

For final dataset and R-code see Supplementary Material. The raw data and R-code for data processing can be provided on request to the senior author.

## Supplementary Materials

The following supporting information can be downloaded at: https://www.mdpi.com/article/10.3390/ani14111641/s1. Figure S1: Satellite maps (Google Earth) of trial site; Table S1: Tasmanian pademelon roadkill counts by periods and sections with MBACI coding used to fit Poisson and NB GLMs (excluding wash-out periods); Table S2: Results of the quasi-Poisson GLM fit to MBACI trial Tasmanian pademelon roadkill counts quantifying the effect of the VF using LORP estimates; R-code and output.
