# Supplementary material for: A Trial of a Virtual Fence to Mitigate Roadkill on an Unsealed Rural Road in Tasmania, Australia
Source: Animals (Basel). 2024 May 31;14(11):1641. doi: 10.3390/ani14111641 (PMC11171368; doi:10.3390/ani14111641)
Supplement: Supplementary file 1 [file animals-14-01641-s001.zip › animals-2956331-supplementary.pdf]

Figure S1. Satellite maps (Google Earth) of trial site.

Start (Section Post 1) and End (Section Post 8) of Virtual Fence installation (large blue placemarks). Intermediate Section Posts 2 to 7 (small placemarks). Section Posts delimiting 7 sections of road length approximately 750 m (see Figure 1 and Table S1) with red Section Posts 3 and 4 delimiting the short (450 m) unfenced Section 3. Location of trial in southern Tasmania shown on bottom panel.

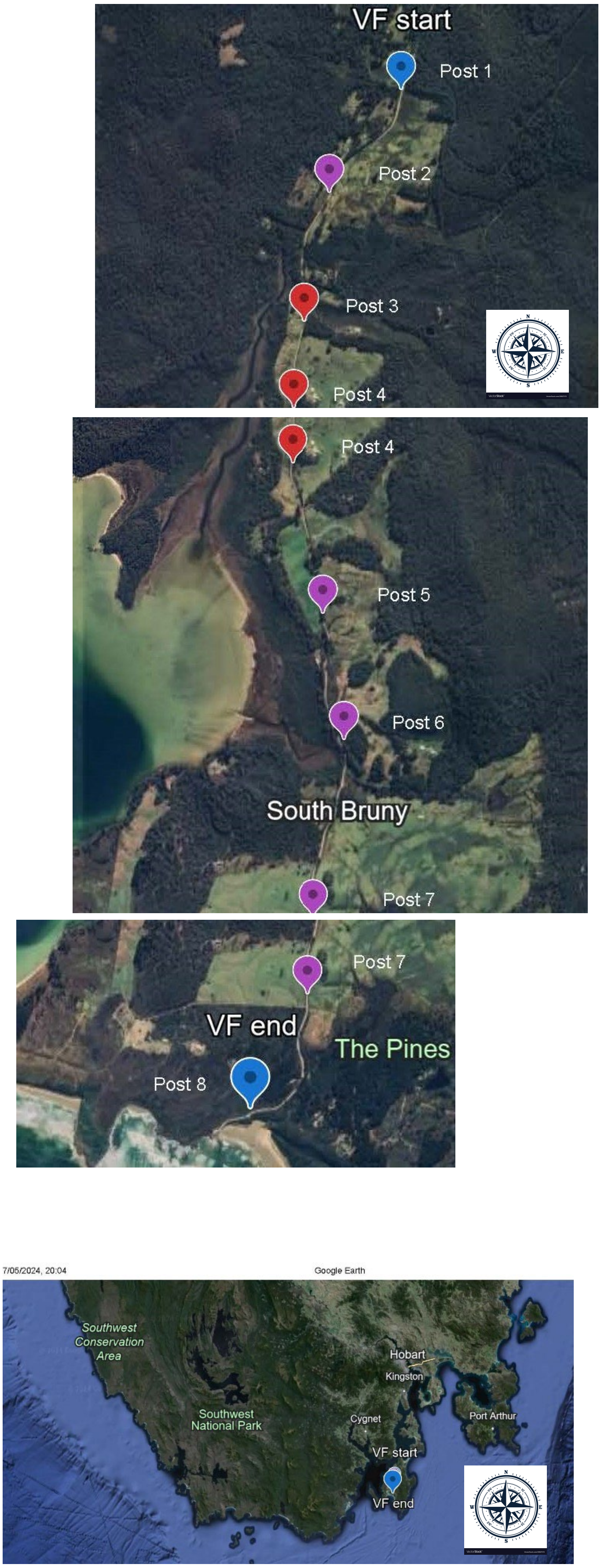

Table S1. Tasmanian Pademelon roadkill counts by periods and sections with MBACI coding used to fit Poisson and NB GLMs (excluding wash-out periods). (`"S2Table.data.countsPad.csv"` in Rcode below).

Unit	Section	Section_len	Block	Rep	Period	Days	VF_status	BACI_code	TP_Counts
1	1	760	1	1	1	136	VF_off	IB	16
2	2	722	1	1	1	136	VF_off	CB	9
3	3	447	1	1	1	136	VF_off	CB	12
4	4	775	1	2	1	136	VF_off	IB	13
5	5	781	1	2	1	136	VF_off	CB	6
6	6	767	1	3	1	136	VF_off	IB	7
7	7	666	1	3	1	136	VF_off	CB	6
8	1	760	1	1	2	70	VF_off	IB	0
9	2	722	1	1	2	70	VF_off	CB	3
10	3	447	1	1	2	70	VF_off	CB	1
11	4	775	1	2	2	70	VF_off	IB	3
12	5	781	1	2	2	70	VF_off	CB	2
13	6	767	1	3	2	70	VF_off	IB	1
14	7	666	1	3	2	70	VF_off	CB	5
15	1	760	1	1	3	120	VF_on	IA	14
16	2	722	1	1	3	120	VF_off	CA	9
17	3	447	1	1	3	120	VF_off	CA	5
18	4	775	1	2	3	120	VF_on	IA	8
19	5	781	1	2	3	120	VF_off	CA	11
20	6	767	1	3	3	120	VF_on	IA	13
21	7	666	1	3	3	120	VF_off	CA	7
22	1	760	2	1	5	101	VF_off	CA	11
23	2	722	2	1	5	101	VF_on	IA	9
24	3	447	2	1	5	101	VF_off	CA	3
25	4	775	2	2	5	101	VF_off	CA	10
26	5	781	2	2	5	101	VF_on	IA	6
27	6	767	2	3	5	101	VF_off	CA	7
28	7	666	2	3	5	101	VF_on	IA	5
29	1	760	2	1	7	126	VF_off	CB	2
30	2	722	2	1	7	126	VF_off	IB	5
31	3	447	2	1	7	126	VF_off	CB	4
32	4	775	2	2	7	126	VF_off	CB	0
33	5	781	2	2	7	126	VF_off	IB	4
34	6	767	2	3	7	126	VF_off	CB	3
35	7	666	2	3	7	126	VF_off	IB	2

Table S2. Results of the quasi-Poisson GLM fit to MBACI trial Tasmanian Pademelon roadkill counts quantifying the effect of the VF using LORP estimates.

Fixed effect	LORP estimates (SE[1], t-value)			Average LORP (SE[2], t-value)	Percentage rate reduction	Poisson deviance,DF
	1	2	3			
Rep	0.199 (0.399, 0.62^{ns})	-1.323 (0.542, -2.44*)	0.610 (0.546, 1.12^{ns})	-0.171 (0.437,-0.39^{ns})	15.7	52.5***, 23
Block	0.185 (0.328, 0.57^{ns})	-0.639 (0.533, -1.20^{ns})		-0.227 (0.416,-0.55 ^{ns})	20.3	47.7**, 27

^{ns} P>0.05, * P <0.05, ** P <0.01, *** P <0.001 (one-sided tests).

1. Unadjusted for over-dispersion
2. Adjusted for over-dispersion using scaling (unadjusted SE)* $\sqrt{deviance / DF}$

R-code

```
### Virtual Fence - Bruny Island Trial ###

### Rcoding : SG Candy March 2024

### read count data for Tasmanian pademelon VF trial
## analyse MBACI data as Poisson (quasi-Poisson) and NB GLMs
## (see Candy and Englefield, 2022, DOI: 10.9734/CJAST/2022/v41i333946 for
## Poisson GLM methods)

library(lattice)
library(mgcv)


data.countsPadNoWout <- read.csv(file="S2Table.data.countsPad.csv", head=T)
names(data.countsPadNoWout)
summary(data.countsPadNoWout)
data.countsPadNoWout$Section <- as.factor(data.countsPadNoWout$Section)
data.countsPadNoWout$Block <- as.factor(data.countsPadNoWout$Block)
data.countsPadNoWout$Rep <- as.factor(data.countsPadNoWout$Rep)
data.countsPadNoWout$Period <- as.factor(data.countsPadNoWout$Period)
data.countsPadNoWout$VF_status <- as.factor(data.countsPadNoWout$VF_status)
data.countsPadNoWout$BACI_code <- as.factor(data.countsPadNoWout$BACI_code)

data.countsPadNoWout$log_months <- log(data.countsPadNoWout$Days/30)
data.countsPadNoWout$log_lengths <- log(data.countsPadNoWout$Section_len/1000)

data.countsPadNoWout$offset <- data.countsPadNoWout$log_months +
                                data.countsPadNoWout$log_lengths

summary(data.countsPadNoWout)


sum(data.countsPadNoWout$TP_Counts)
tapply(X=data.countsPadNoWout$TP_Counts, INDEX=list(data.countsPadNoWout$BACI_code,
    data.countsPadNoWout$Block), FUN=sum)

Interact_50pc <- log(0.5)

BvsA <- as.integer(data.countsPadNoWout$BACI_code %in% c("IB","CB"))+
           2*as.integer(data.countsPadNoWout$BACI_code %in% c("IA","CA"))

data.countsPadNoWout$BvsA_f <- factor(x=BvsA, levels=c(1:2), labels=c("Before","After"))

CvsI <- as.integer(data.countsPadNoWout$BACI_code %in% c("CB","CA"))+
           2*as.integer(data.countsPadNoWout$BACI_code %in% c("IB","IA"))

data.countsPadNoWout$CvsI_f <- factor(x=CvsI, levels=c(1:2), labels=c("Control","Impact"))

#write.csv(file="data.countsPadNoWout.csv", x=data.countsPadNoWout)

## raw rates using all data

counts <- tapply(X=data.countsPadNoWout$TP_Counts,
      INDEX=list(data.countsPadNoWout$VF_status), FUN=sum)
dayLen <- data.countsPadNoWout$Days*data.countsPadNoWout$Section_len

daysL_T <- tapply(X=dayLen,
```

```
      INDEX=list(data.countsPadNoWout$VF_status), FUN=sum)
rates <- 30*1000*counts/daysL_T

print(counts)
print(rates)

## raw rates using matched off-on, on-off sections (periods 3 and 5)

data.countsPadNoWoutM <- data.countsPadNoWout[data.countsPadNoWout$Period %in%
       c("3","5") & data.countsPadNoWout$Section!="3",]

summary(data.countsPadNoWoutM)

print(data.countsPadNoWoutM)

counts <- tapply(X=data.countsPadNoWoutM$TP_Counts,
      INDEX=list(data.countsPadNoWoutM$VF_status), FUN=sum)
dayLen <- data.countsPadNoWoutM$Days*data.countsPadNoWoutM$Section_len

daysL_T <- tapply(X=dayLen,
      INDEX=list(data.countsPadNoWoutM$VF_status), FUN=sum)
rates <- 30*1000*counts/daysL_T

print(counts)
print(rates)

glm.baci.02          <-          glm(formula=TP_Counts          ~          Block:Rep          +
Block:Rep:BvsA+Block:Rep:CvsI+Block:Rep:BvsA:CvsI,
          offset=offset, family=poisson, data=data.countsPadNoWout)

summary(glm.baci.02)

glm.baci.02 <- glm(formula=TP_Counts ~ Rep + Rep:BvsA+Rep:CvsI+Rep:BvsA:CvsI,
          offset=offset, family=poisson, data=data.countsPadNoWout)

summary(glm.baci.02)

c(glm.baci.02$deviance,glm.baci.02$df.residual,qchisq(p=0.05,    df=glm.baci.02$df.residual,
ncp = 0,
                  lower.tail = FALSE, log.p = FALSE))

length(glm.baci.02$coefficients)


vec <- matrix(data=rep(1,3), nrow=1, ncol=3)

Interact_pars <- sum(glm.baci.02$coefficients[10:12])/3
Interact_pars_SE <- (vec %*% (vcov(glm.baci.02)[10:12,10:12])) %*% t(vec)
## use quasi-Poisson for averaged over "Rep" effect see Table S1 Supplementary Material)
Interact_pars_SE                                                                        <-
(((glm.baci.02$deviance/glm.baci.02$df.residual)*Interact_pars_SE)^0.5)/3

phi <- glm.baci.02$deviance/glm.baci.02$df.residual
CritH0 <- qnorm(p=0.05, mean = 0, sd = Interact_pars_SE, lower.tail = TRUE, log.p = FALSE)
# calcuate Prob(Type II error) for a true reduction of
PrTypeII <- pnorm(q=CritH0, mean =Interact_50pc, sd = Interact_pars_SE, lower.tail = FALSE,
log.p = FALSE)
Power <- 1-PrTypeII
Interact_R <- 100*(1-exp(Interact_pars))
```

```
t_stat <- Interact_pars/Interact_pars_SE
print(c(Interact_pars,Interact_pars_SE,t_stat,Interact_R,CritH0,PrTypeII,Power,phi))


glm.baci.03 <- glm(formula=TP_Counts ~ Block + Block:BvsA+Block:CvsI+Block:BvsA:CvsI,
          offset=offset, family=poisson, data=data.countsPadNoWout)

summary(glm.baci.03)

c(glm.baci.03$deviance,glm.baci.03$df.residual,qchisq(p=0.05,    df=glm.baci.03$df.residual,
ncp = 0,
                  lower.tail = FALSE, log.p = FALSE))


vec <- matrix(data=rep(1,2), nrow=1, ncol=2)

Interact_pars <- sum(glm.baci.03$coefficients[7:8])/2
Interact_pars_SE <- (vec %*% (vcov(glm.baci.03)[7:8,7:8])) %*% t(vec)
## use quasi-Poisson for averaged over "Block" effect see Table S1 Supplementary Material)
Interact_pars_SE                                                                        <-
(((glm.baci.03$deviance/glm.baci.03$df.residual)*Interact_pars_SE)^0.5)/2
phi <- glm.baci.03$deviance/glm.baci.03$df.residual

CritH0 <- qnorm(p=0.05, mean = 0, sd = Interact_pars_SE, lower.tail = TRUE, log.p = FALSE)
# calcuate Prob(Type II error) for a true reduction of
PrTypeII <- pnorm(q=CritH0, mean =Interact_50pc, sd = Interact_pars_SE, lower.tail = FALSE,
log.p = FALSE)
Power <- 1-PrTypeII
Interact_R <- 100*(1-exp(Interact_pars))
t_stat <- Interact_pars/Interact_pars_SE
print(c(Interact_pars,Interact_pars_SE,t_stat,Interact_R,CritH0,PrTypeII,Power,phi))

## now try NB (see Table 1)

gam.baci.02         <-         gam(formula=TP_Counts         ~         Block:Rep         +
Block:Rep:BvsA+Block:Rep:CvsI+Block:Rep:BvsA:CvsI,
          offset=offset, family=nb(link="log"), data=data.countsPadNoWout)

summary(gam.baci.02)

gam.baci.02 <- gam(formula=TP_Counts ~ Rep + Rep:BvsA+Rep:CvsI+Rep:BvsA:CvsI,
          offset=offset, family=nb(link="log"), data=data.countsPadNoWout)

summary(gam.baci.02)

c(gam.baci.02$deviance,gam.baci.02$df.residual,qchisq(p=0.05,    df=gam.baci.02$df.residual,
ncp = 0,
                  lower.tail = FALSE, log.p = FALSE))

vec <- matrix(data=rep(1,3), nrow=1, ncol=3)

Interact_pars <- sum(gam.baci.02$coefficients[10:12])/3
Interact_pars_SE <- (vec %*% (vcov(gam.baci.02)[10:12,10:12])) %*% t(vec)
phi <- 1
Interact_pars_SE <- ((phi*Interact_pars_SE)^0.5)/3

gam.baci.02$family$getTheta(TRUE)
phi <- gam.baci.02$deviance/gam.baci.02$df.residual
```

```
CritH0 <- qnorm(p=0.05, mean = 0, sd = Interact_pars_SE, lower.tail = TRUE, log.p = FALSE)
# calcuate Prob(Type II error) for a true reduction of
PrTypeII <- pnorm(q=CritH0, mean =Interact_50pc, sd = Interact_pars_SE, lower.tail = FALSE,
log.p = FALSE)
Power <- 1-PrTypeII
Interact_R <- 100*(1-exp(Interact_pars))

t_stat <- Interact_pars/Interact_pars_SE
print(c(Interact_pars,Interact_pars_SE,t_stat,Interact_R,CritH0,PrTypeII,Power,phi))


gam.baci.03 <- gam(formula=TP_Counts ~ Block + Block:BvsA+Block:CvsI+Block:BvsA:CvsI,
          offset=offset, family=nb(link="log"), data=data.countsPadNoWout)

summary(gam.baci.03)

c(gam.baci.03$deviance,gam.baci.03$df.residual,qchisq(p=0.05,    df=gam.baci.03$df.residual,
ncp = 0,
                  lower.tail = FALSE, log.p = FALSE))


vec <- matrix(data=rep(1,2), nrow=1, ncol=2)

Interact_pars <- sum(gam.baci.03$coefficients[7:8])/2
Interact_pars_SE <- (vec %*% (vcov(gam.baci.03)[7:8,7:8])) %*% t(vec)
phi <- 1
Interact_pars_SE <- (((phi)*Interact_pars_SE)^0.5)/2
gam.baci.03$family$getTheta(TRUE)
phi <- gam.baci.03$deviance/gam.baci.03$df.residual

CritH0 <- qnorm(p=0.05, mean = 0, sd = Interact_pars_SE, lower.tail = TRUE, log.p = FALSE)
# calcuate Prob(Type II error) for a true reduction of
PrTypeII <- pnorm(q=CritH0, mean =Interact_50pc, sd = Interact_pars_SE, lower.tail = FALSE,
log.p = FALSE)
Power <- 1-PrTypeII
Interact_R <- 100*(1-exp(Interact_pars))
t_stat <- Interact_pars/Interact_pars_SE
print(c(Interact_pars,Interact_pars_SE,t_stat,Interact_R,CritH0,PrTypeII,Power,phi))
```

R-code output

```
R version 4.0.3 (2020-10-10) -- "Bunny-Wunnies Freak Out"
Copyright (C) 2020 The R Foundation for Statistical Computing
Platform: x86_64-w64-mingw32/x64 (64-bit)

R is free software and comes with ABSOLUTELY NO WARRANTY.
You are welcome to redistribute it under certain conditions.
Type 'license()' or 'licence()' for distribution details.

  Natural language support but running in an English locale

R is a collaborative project with many contributors.
Type 'contributors()' for more information and
'citation()' on how to cite R or R packages in publications.

Type 'demo()' for some demos, 'help()' for on-line help, or
'help.start()' for an HTML browser interface to help.
Type 'q()' to quit R.
```

```
> ### Virtual Fence - Bruny Island Trial ###
>
> ### Rcoding : SG Candy March 2024
>
> ### read count data for Tasmanian pademelon VF trial
> ## analyse MBACI data as Poisson (quasi-Poisson) and NB GLMs
> ## (see Candy and Englefield, 2022, DOI: 10.9734/CJAST/2022/v41i333946 for
> ## Poisson GLM methods)
>
> library(lattice)
> library(mgcv)
>
>
> data.countsPadNoWout <- read.csv(file="S2Table.data.countsPad.csv", head=T)
> names(data.countsPadNoWout)
 [1] "Unit"        "Section"     "Section_len" "Block"       "Rep"
 [6] "Period"      "Days"        "VF_status"   "BACI_code"   "TP_Counts"
> summary(data.countsPadNoWout)
      Unit         Section   Section_len        Block          Rep
 Min.   : 1.0   Min.   :1   Min.   :447.0   Min.   :1.0   Min.   :1.000
 1st Qu.: 9.5   1st Qu.:2   1st Qu.:666.0   1st Qu.:1.0   1st Qu.:1.000
 Median :18.0   Median :4   Median :760.0   Median :1.0   Median :2.000
 Mean   :18.0   Mean   :4   Mean   :702.6   Mean   :1.4   Mean   :1.857
 3rd Qu.:26.5   3rd Qu.:6   3rd Qu.:775.0   3rd Qu.:2.0   3rd Qu.:3.000
 Max.   :35.0   Max.   :7   Max.   :781.0   Max.   :2.0   Max.   :3.000
     Period          Days        VF_status          BACI_code
 Min.   :1.0   Min.   : 70.0   Length:35          Length:35
 1st Qu.:2.0   1st Qu.:101.0   Class :character   Class :character
 Median :3.0   Median :120.0   Mode  :character   Mode  :character
 Mean   :3.6   Mean   :110.6
 3rd Qu.:5.0   3rd Qu.:126.0
 Max.   :7.0   Max.   :136.0
   TP_Counts
 Min.   : 0.000
 1st Qu.: 3.000
 Median : 6.000
 Mean   : 6.343
 3rd Qu.: 9.000
 Max.   :16.000
> data.countsPadNoWout$Section <- as.factor(data.countsPadNoWout$Section)
> data.countsPadNoWout$Block <- as.factor(data.countsPadNoWout$Block)
> data.countsPadNoWout$Rep <- as.factor(data.countsPadNoWout$Rep)
> data.countsPadNoWout$Period <- as.factor(data.countsPadNoWout$Period)
> data.countsPadNoWout$VF_status <- as.factor(data.countsPadNoWout$VF_status)
> data.countsPadNoWout$BACI_code <- as.factor(data.countsPadNoWout$BACI_code)
>
> data.countsPadNoWout$log_months <- log(data.countsPadNoWout$Days/30)
> data.countsPadNoWout$log_lengths <- log(data.countsPadNoWout$Section_len/1000)
>
> data.countsPadNoWout$offset <- data.countsPadNoWout$log_months +
+                                  data.countsPadNoWout$log_lengths
>
> summary(data.countsPadNoWout)
      Unit       Section  Section_len    Block  Rep    Period      Days
 Min.   : 1.0   1:5     Min.   :447.0   1:21   1:15   1:7    Min.   : 70.0
 1st Qu.: 9.5   2:5     1st Qu.:666.0   2:14   2:10   2:7    1st Qu.:101.0
 Median :18.0   3:5     Median :760.0          3:10   3:7    Median :120.0
 Mean   :18.0   4:5     Mean   :702.6                 5:7    Mean   :110.6
```

```
 3rd Qu.:26.5   5:5     3rd Qu.:775.0                 7:7    3rd Qu.:126.0
 Max.   :35.0   6:5     Max.   :781.0                        Max.   :136.0
                7:5
  VF_status  BACI_code   TP_Counts        log_months      log_lengths
 VF_off:29   CA: 8     Min.   : 0.000   Min.   :0.8473   Min.   :-0.8052
 VF_on : 6   CB:12     1st Qu.: 3.000   1st Qu.:1.2139   1st Qu.:-0.4065
             IA: 6     Median : 6.000   Median :1.3863   Median :-0.2744
             IB: 9     Mean   : 6.343   Mean   :1.2788   Mean   :-0.3685
                       3rd Qu.: 9.000   3rd Qu.:1.4351   3rd Qu.:-0.2549
                       Max.   :16.000   Max.   :1.5115   Max.   :-0.2472

     offset
 Min.   :0.0421
 1st Qu.:0.6150
 Median :0.9798
 Mean   :0.9104
 3rd Qu.:1.1499
 Max.   :1.2643

>
>
> sum(data.countsPadNoWout$TP_Counts)
[1] 222
> tapply(X=data.countsPadNoWout$TP_Counts, INDEX=list(data.countsPadNoWout$BACI_code,
+     data.countsPadNoWout$Block), FUN=sum)
    1  2
CA 32 31
CB 44  9
IA 35 20
IB 40 11
>
> Interact_50pc <- log(0.5)
>
> BvsA <- as.integer(data.countsPadNoWout$BACI_code %in% c("IB","CB"))+
+            2*as.integer(data.countsPadNoWout$BACI_code %in% c("IA","CA"))
>
> data.countsPadNoWout$BvsA_f <- factor(x=BvsA, levels=c(1:2), labels=c("Before","After"))
>
> CvsI <- as.integer(data.countsPadNoWout$BACI_code %in% c("CB","CA"))+
+            2*as.integer(data.countsPadNoWout$BACI_code %in% c("IB","IA"))
>
> data.countsPadNoWout$CvsI_f <- factor(x=CvsI, levels=c(1:2), labels=c("Control","Impact"))
>
> #write.csv(file="data.countsPadNoWout.csv", x=data.countsPadNoWout)
>
> ## raw rates using all data
>
> counts <- tapply(X=data.countsPadNoWout$TP_Counts,
+       INDEX=list(data.countsPadNoWout$VF_status), FUN=sum)
> dayLen <- data.countsPadNoWout$Days*data.countsPadNoWout$Section_len
>
> daysL_T <- tapply(X=dayLen,
+       INDEX=list(data.countsPadNoWout$VF_status), FUN=sum)
> rates <- 30*1000*counts/daysL_T
>
> print(counts)
VF_off  VF_on
   167     55
> print(rates)
```

```
  VF_off    VF_on
2.252348 3.331254
>
> ## raw rates using matched off-on, on-off sections (periods 3 and 5)
>
> data.countsPadNoWoutM <- data.countsPadNoWout[data.countsPadNoWout$Period %in%
+        c("3","5") & data.countsPadNoWout$Section!="3",]
>
> summary(data.countsPadNoWoutM)
      Unit        Section  Section_len    Block Rep   Period      Days
 Min.   :15.00   1:2     Min.   :666.0   1:6   1:4   1:0    Min.   :101.0
 1st Qu.:18.75   2:2     1st Qu.:722.0   2:6   2:4   2:0    1st Qu.:101.0
 Median :21.50   3:0     Median :763.5         3:4   3:6    Median :110.5
 Mean   :21.67   4:2     Mean   :745.2               5:6    Mean   :110.5
 3rd Qu.:25.25   5:2     3rd Qu.:775.0               7:0    3rd Qu.:120.0
 Max.   :28.00   6:2     Max.   :781.0                      Max.   :120.0
                 7:2
  VF_status BACI_code   TP_Counts        log_months     log_lengths
 VF_off:6   CA:6      Min.   : 5.000   Min.   :1.214   Min.   :-0.4065
 VF_on :6   CB:0      1st Qu.: 7.000   1st Qu.:1.214   1st Qu.:-0.3257
            IA:6      Median : 9.000   Median :1.300   Median :-0.2699
            IB:0      Mean   : 9.167   Mean   :1.300   Mean   :-0.2957
                      3rd Qu.:11.000   3rd Qu.:1.386   3rd Qu.:-0.2549
                      Max.   :14.000   Max.   :1.386   Max.   :-0.2472

     offset          BvsA_f       CvsI_f
 Min.   :0.8075   Before: 0   Control:6
 1st Qu.:0.9464   After :12   Impact :6
 Median :0.9733
 Mean   :1.0044
 3rd Qu.:1.1141
 Max.   :1.1391

>
> print(data.countsPadNoWoutM)
   Unit Section Section_len Block Rep Period Days VF_status BACI_code TP_Counts
15   15       1         760     1   1      3  120     VF_on        IA        14
16   16       2         722     1   1      3  120    VF_off        CA         9
18   18       4         775     1   2      3  120     VF_on        IA         8
19   19       5         781     1   2      3  120    VF_off        CA        11
20   20       6         767     1   3      3  120     VF_on        IA        13
21   21       7         666     1   3      3  120    VF_off        CA         7
22   22       1         760     2   1      5  101    VF_off        CA        11
23   23       2         722     2   1      5  101     VF_on        IA         9
25   25       4         775     2   2      5  101    VF_off        CA        10
26   26       5         781     2   2      5  101     VF_on        IA         6
27   27       6         767     2   3      5  101    VF_off        CA         7
28   28       7         666     2   3      5  101     VF_on        IA         5
   log_months log_lengths    offset BvsA_f  CvsI_f
15   1.386294  -0.2744368 1.1118575  After  Impact
16   1.386294  -0.3257301 1.0605642  After Control
18   1.386294  -0.2548922 1.1314021  After  Impact
19   1.386294  -0.2471801 1.1391142  After Control
20   1.386294  -0.2652685 1.1210259  After  Impact
21   1.386294  -0.4064656 0.9798288  After Control
22   1.213923  -0.2744368 0.9394863  After Control
23   1.213923  -0.3257301 0.8881930  After  Impact
25   1.213923  -0.2548922 0.9590309  After Control
26   1.213923  -0.2471801 0.9667430  After  Impact
```

```
27   1.213923  -0.2652685 0.9486547  After Control
28   1.213923  -0.4064656 0.8074575  After  Impact
>
> counts <- tapply(X=data.countsPadNoWoutM$TP_Counts,
+       INDEX=list(data.countsPadNoWoutM$VF_status), FUN=sum)
> dayLen <- data.countsPadNoWoutM$Days*data.countsPadNoWoutM$Section_len
>
> daysL_T <- tapply(X=dayLen,
+       INDEX=list(data.countsPadNoWoutM$VF_status), FUN=sum)
> rates <- 30*1000*counts/daysL_T
>
> print(counts)
VF_off  VF_on
    55     55
> print(rates)
  VF_off    VF_on
3.348337 3.331254
>
>       glm.baci.02       <-       glm(formula=TP_Counts       ~       Block:Rep       +
Block:Rep:BvsA+Block:Rep:CvsI+Block:Rep:BvsA:CvsI,
+           offset=offset, family=poisson, data=data.countsPadNoWout)
>
> summary(glm.baci.02)

Call:
glm(formula = TP_Counts ~ Block:Rep + Block:Rep:BvsA + Block:Rep:CvsI +
    Block:Rep:BvsA:CvsI, family = poisson, data = data.countsPadNoWout,
    offset = offset)

Deviance Residuals:
    Min       1Q   Median       3Q      Max
-3.2975  -0.4195   0.0000   0.0597   2.0111

Coefficients: (1 not defined because of singularities)
                        Estimate Std. Error z value Pr(>|z|)
(Intercept)             -0.80640    2.84689  -0.283   0.7770
Block1:Rep1              2.44387    3.05809   0.799   0.4242
Block2:Rep1             -0.68458    3.45952  -0.198   0.8431
Block1:Rep2             -1.36495    3.29293  -0.415   0.6785
Block2:Rep2            -71.38358 8413.45068  -0.008   0.9932
Block1:Rep3              2.99283    3.27226   0.915   0.3604
Block2:Rep3                   NA         NA      NA       NA
Block1:Rep1:BvsA        -0.48572    0.76134  -0.638   0.5235
Block2:Rep1:BvsA         1.32797    1.12405   1.181   0.2374
Block1:Rep2:BvsA         1.87044    1.02525   1.824   0.0681 .
Block2:Rep2:BvsA        37.02604 4206.72515   0.009   0.9930
Block1:Rep3:BvsA        -0.84909    1.06630  -0.796   0.4259
Block2:Rep3:BvsA         0.99947    1.61393   0.619   0.5357
Block1:Rep1:CvsI        -0.46199    0.74354  -0.621   0.5344
Block2:Rep1:CvsI         0.59106    1.28421   0.460   0.6453
Block1:Rep2:CvsI         1.71246    0.98281   1.742   0.0814 .
Block2:Rep2:CvsI        35.88087 4206.72521   0.009   0.9932
Block1:Rep3:CvsI        -1.39714    1.04087  -1.342   0.1795
Block2:Rep3:CvsI        -0.33326    1.91734  -0.174   0.8620
Block1:Rep1:BvsA:CvsI    0.44629    0.49534   0.901   0.3676
Block2:Rep1:BvsA:CvsI   -0.25951    0.74108  -0.350   0.7262
Block1:Rep2:BvsA:CvsI   -1.01160    0.63514  -1.593   0.1112
Block2:Rep2:BvsA:CvsI  -18.19971 2103.36265  -0.009   0.9931
Block1:Rep3:BvsA:CvsI    0.93749    0.66007   1.420   0.1555
```

```
Block2:Rep3:BvsA:CvsI    0.06899    1.08452   0.064   0.9493
---
Signif. codes:  0  ***  0.001  **  0.01  *  0.05  .  0.1     1

(Dispersion parameter for poisson family taken to be 1)

    Null deviance: 83.048  on 34  degrees of freedom
Residual deviance: 29.237  on 11  degrees of freedom
AIC: 194.93

Number of Fisher Scoring iterations: 14

>
> glm.baci.02 <- glm(formula=TP_Counts ~ Rep + Rep:BvsA+Rep:CvsI+Rep:BvsA:CvsI,
+            offset=offset, family=poisson, data=data.countsPadNoWout)
>
> summary(glm.baci.02)

Call:
glm(formula = TP_Counts ~ Rep + Rep:BvsA + Rep:CvsI + Rep:BvsA:CvsI,
    family = poisson, data = data.countsPadNoWout, offset = offset)

Deviance Residuals:
    Min       1Q   Median       3Q      Max
-3.0044  -0.9055  -0.1318   0.3872   2.7570

Coefficients:
               Estimate Std. Error z value Pr(>|z|)
(Intercept)      0.6856     0.9450   0.726  0.46813
Rep2            -4.3737     1.8316  -2.388  0.01694 *
Rep3             0.4842     1.6665   0.291  0.77139
Rep1:BvsA        0.1035     0.6025   0.172  0.86359
Rep2:BvsA        2.6956     0.9011   2.992  0.00278 **
Rep3:BvsA       -0.2137     0.8526  -0.251  0.80208
Rep1:CvsI       -0.1267     0.6314  -0.201  0.84102
Rep2:CvsI        2.2411     0.9050   2.476  0.01327 *
Rep3:CvsI       -0.9801     0.9015  -1.087  0.27696
Rep1:BvsA:CvsI   0.1992     0.3988   0.499  0.61746
Rep2:BvsA:CvsI  -1.3230     0.5423  -2.440  0.01470 *
Rep3:BvsA:CvsI   0.6096     0.5463   1.116  0.26442
---
Signif. codes:  0  ***  0.001  **  0.01  *  0.05  .  0.1     1

(Dispersion parameter for poisson family taken to be 1)

    Null deviance: 83.048  on 34  degrees of freedom
Residual deviance: 52.546  on 23  degrees of freedom
AIC: 194.24

Number of Fisher Scoring iterations: 5

>
>  c(glm.baci.02$deviance,glm.baci.02$df.residual,qchisq(p=0.05,  df=glm.baci.02$df.residual,
ncp = 0,
+                    lower.tail = FALSE, log.p = FALSE))
[1] 52.54601 23.00000 35.17246
>
> length(glm.baci.02$coefficients)
[1] 12
```

```
>
>
> vec <- matrix(data=rep(1,3), nrow=1, ncol=3)
>
> Interact_pars <- sum(glm.baci.02$coefficients[10:12])/3
> Interact_pars_SE <- (vec %*% (vcov(glm.baci.02)[10:12,10:12])) %*% t(vec)
> ## use quasi-Poisson for averaged over "Rep" effect see Table S1 Supplementary Material)
>                                 Interact_pars_SE                              <-
(((glm.baci.02$deviance/glm.baci.02$df.residual)*Interact_pars_SE)^0.5)/3
>
> phi <- glm.baci.02$deviance/glm.baci.02$df.residual
> CritH0 <- qnorm(p=0.05, mean = 0, sd = Interact_pars_SE, lower.tail = TRUE, log.p = FALSE)
> # calcuate Prob(Type II error) for a true reduction of
> PrTypeII <- pnorm(q=CritH0, mean =Interact_50pc, sd = Interact_pars_SE, lower.tail = FALSE,
log.p = FALSE)
> Power <- 1-PrTypeII
> Interact_R <- 100*(1-exp(Interact_pars))
>
> t_stat <- Interact_pars/Interact_pars_SE
> print(c(Interact_pars,Interact_pars_SE,t_stat,Interact_R,CritH0,PrTypeII,Power,phi))
[1] -0.1713720  0.4367749 -0.3923577 15.7491907 -0.7184308  0.5230807  0.4769193
[8]  2.2846092
>
>
> glm.baci.03 <- glm(formula=TP_Counts ~ Block + Block:BvsA+Block:CvsI+Block:BvsA:CvsI,
+           offset=offset, family=poisson, data=data.countsPadNoWout)
>
> summary(glm.baci.03)

Call:
glm(formula = TP_Counts ~ Block + Block:BvsA + Block:CvsI + Block:BvsA:CvsI,
    family = poisson, data = data.countsPadNoWout, offset = offset)

Deviance Residuals:
    Min       1Q   Median       3Q      Max
-2.9958  -0.7992  -0.3205   0.5201   2.6672

Coefficients:
                 Estimate Std. Error z value Pr(>|z|)
(Intercept)       0.82630    0.78563   1.052   0.2929
Block2           -3.60989    1.71396  -2.106   0.0352 *
Block1:BvsA       0.03701    0.51912   0.071   0.9432
Block2:BvsA       2.09685    0.84521   2.481   0.0131 *
Block1:CvsI      -0.15236    0.50073  -0.304   0.7609
Block2:CvsI       1.07657    0.94358   1.141   0.2539
Block1:BvsA:CvsI  0.18492    0.32795   0.564   0.5728
Block2:BvsA:CvsI -0.63893    0.53318  -1.198   0.2308
---
Signif. codes:  0  ***  0.001  **  0.01  *  0.05  .  0.1     1

(Dispersion parameter for poisson family taken to be 1)

    Null deviance: 83.048  on 34  degrees of freedom
Residual deviance: 47.669  on 27  degrees of freedom
AIC: 181.36

Number of Fisher Scoring iterations: 5

>
```

```
>  c(glm.baci.03$deviance,glm.baci.03$df.residual,qchisq(p=0.05,  df=glm.baci.03$df.residual,
ncp = 0,
+                    lower.tail = FALSE, log.p = FALSE))
[1] 47.66938 27.00000 40.11327
>
>
> vec <- matrix(data=rep(1,2), nrow=1, ncol=2)
>
> Interact_pars <- sum(glm.baci.03$coefficients[7:8])/2
> Interact_pars_SE <- (vec %*% (vcov(glm.baci.03)[7:8,7:8])) %*% t(vec)
> ## use quasi-Poisson for averaged over "Block" effect see Table S1 Supplementary Material)
>                                     Interact_pars_SE                                  <-
(((glm.baci.03$deviance/glm.baci.03$df.residual)*Interact_pars_SE)^0.5)/2
> phi <- glm.baci.03$deviance/glm.baci.03$df.residual
>
> CritH0 <- qnorm(p=0.05, mean = 0, sd = Interact_pars_SE, lower.tail = TRUE, log.p = FALSE)
> # calcuate Prob(Type II error) for a true reduction of
> PrTypeII <- pnorm(q=CritH0, mean =Interact_50pc, sd = Interact_pars_SE, lower.tail = FALSE,
log.p = FALSE)
> Power <- 1-PrTypeII
> Interact_R <- 100*(1-exp(Interact_pars))
> t_stat <- Interact_pars/Interact_pars_SE
> print(c(Interact_pars,Interact_pars_SE,t_stat,Interact_R,CritH0,PrTypeII,Power,phi))
[1] -0.2270016  0.4158674 -0.5458511 20.3080528 -0.6840409  0.4912651  0.5087349
[8]  1.7655326
>
> ## now try NB (see Table 1)
>
>       gam.baci.02       <-       gam(formula=TP_Counts       ~       Block:Rep       +
Block:Rep:BvsA+Block:Rep:CvsI+Block:Rep:BvsA:CvsI,
+            offset=offset, family=nb(link="log"), data=data.countsPadNoWout)
Warning message:
In newton(lsp = lsp, X = G$X, y = G$y, Eb = G$Eb, UrS = G$UrS, L = G$L,  :
  Fitting terminated with step failure - check results carefully
>
> summary(gam.baci.02)

Family: Negative Binomial(2.795)
Link function: log

Formula:
TP_Counts ~ Block:Rep + Block:Rep:BvsA + Block:Rep:CvsI + Block:Rep:BvsA:CvsI

Parametric coefficients:
                        Estimate Std. Error z value Pr(>|z|)
(Intercept)           -4.087e+02  2.684e+08   0.000    1.000
Block1:Rep1            4.105e+02  2.684e+08   0.000    1.000
Block2:Rep1            4.077e+02  2.684e+08   0.000    1.000
Block1:Rep2            4.066e+02  2.684e+08   0.000    1.000
Block2:Rep2            0.000e+00  0.000e+00      NA       NA
Block1:Rep3            4.113e+02  2.684e+08   0.000    1.000
Block2:Rep3            4.079e+02  2.684e+08   0.000    1.000
Block1:Rep1:BvsA      -5.954e-01  1.500e+00  -0.397    0.691
Block2:Rep1:BvsA       1.040e+00  1.848e+00   0.563    0.573
Block1:Rep2:BvsA       1.863e+00  1.948e+00   0.956    0.339
Block2:Rep2:BvsA       2.053e+02  1.342e+08   0.000    1.000
Block1:Rep3:BvsA      -1.040e+00  1.961e+00  -0.530    0.596
Block2:Rep3:BvsA       9.995e-01  2.486e+00   0.402    0.688
Block1:Rep1:CvsI      -7.437e-01  1.505e+00  -0.494    0.621
```

```
Block2:Rep1:CvsI       3.649e-01  2.076e+00   0.176    0.860
Block1:Rep2:CvsI       1.626e+00  1.791e+00   0.908    0.364
Block2:Rep2:CvsI       2.041e+02  1.342e+08   0.000    1.000
Block1:Rep3:CvsI      -1.674e+00  1.821e+00  -0.919    0.358
Block2:Rep3:CvsI      -3.333e-01  2.693e+00  -0.124    0.902
Block1:Rep1:BvsA:CvsI  5.916e-01  1.039e+00   0.570    0.569
Block2:Rep1:BvsA:CvsI -1.157e-01  1.274e+00  -0.091    0.928
Block1:Rep2:BvsA:CvsI -9.681e-01  1.225e+00  -0.790    0.429
Block2:Rep2:BvsA:CvsI -1.023e+02  6.711e+07   0.000    1.000
Block1:Rep3:BvsA:CvsI  1.076e+00  1.237e+00   0.870    0.384
Block2:Rep3:BvsA:CvsI  6.899e-02  1.615e+00   0.043    0.966


Rank: 24/25
R-sq.(adj) =  0.189   Deviance explained = 59.7%
-REML = 54.078  Scale est. = 1         n = 35
>
> gam.baci.02 <- gam(formula=TP_Counts ~ Rep + Rep:BvsA+Rep:CvsI+Rep:BvsA:CvsI,
+           offset=offset, family=nb(link="log"), data=data.countsPadNoWout)
>
> summary(gam.baci.02)

Family: Negative Binomial(4.845)
Link function: log

Formula:
TP_Counts ~ Rep + Rep:BvsA + Rep:CvsI + Rep:BvsA:CvsI

Parametric coefficients:
               Estimate Std. Error z value Pr(>|z|)
(Intercept)     0.97295    1.44857   0.672   0.5018
Rep2           -4.61987    2.55511  -1.808   0.0706 .
Rep3            0.49196    2.42792   0.203   0.8394
Rep1:BvsA      -0.06196    0.95096  -0.065   0.9480
Rep2:BvsA       2.67907    1.30069   2.060   0.0394 *
Rep3:BvsA      -0.34624    1.26255  -0.274   0.7839
Rep1:CvsI      -0.36267    1.00284  -0.362   0.7176
Rep2:CvsI       2.19102    1.26774   1.728   0.0839 .
Rep3:CvsI      -1.15886    1.26469  -0.916   0.3595
Rep1:BvsA:CvsI  0.32600    0.65520   0.498   0.6188
Rep2:BvsA:CvsI -1.30069    0.80397  -1.618   0.1057
Rep3:BvsA:CvsI  0.68406    0.80737   0.847   0.3968
---
Signif. codes:  0  ***  0.001  **  0.01  *  0.05  .  0.1     1


R-sq.(adj) =  0.315   Deviance explained = 32.1%
-REML = 87.343  Scale est. = 1         n = 35
>
>  c(gam.baci.02$deviance,gam.baci.02$df.residual,qchisq(p=0.05,  df=gam.baci.02$df.residual,
ncp = 0,
+                    lower.tail = FALSE, log.p = FALSE))
[1] 27.87874 23.00000 35.17246
>
> vec <- matrix(data=rep(1,3), nrow=1, ncol=3)
>
> Interact_pars <- sum(gam.baci.02$coefficients[10:12])/3
> Interact_pars_SE <- (vec %*% (vcov(gam.baci.02)[10:12,10:12])) %*% t(vec)
> phi <- 1
```

```
> Interact_pars_SE <- ((phi*Interact_pars_SE)^0.5)/3
>
> gam.baci.02$family$getTheta(TRUE)
[1] 4.844701
> phi <- gam.baci.02$deviance/gam.baci.02$df.residual
>
> CritH0 <- qnorm(p=0.05, mean = 0, sd = Interact_pars_SE, lower.tail = TRUE, log.p = FALSE)
> # calcuate Prob(Type II error) for a true reduction of
> PrTypeII <- pnorm(q=CritH0, mean =Interact_50pc, sd = Interact_pars_SE, lower.tail = FALSE,
log.p = FALSE)
> Power <- 1-PrTypeII
> Interact_R <- 100*(1-exp(Interact_pars))
>
> t_stat <- Interact_pars/Interact_pars_SE
> print(c(Interact_pars,Interact_pars_SE,t_stat,Interact_R,CritH0,PrTypeII,Power,phi))
[1] -0.0968789  0.4381150 -0.2211267  9.2334082 -0.7206350  0.5250136  0.4749864
[8]  1.2121191
>
>
> gam.baci.03 <- gam(formula=TP_Counts ~ Block + Block:BvsA+Block:CvsI+Block:BvsA:CvsI,
+           offset=offset, family=nb(link="log"), data=data.countsPadNoWout)
>
> summary(gam.baci.03)

Family: Negative Binomial(11.579)
Link function: log

Formula:
TP_Counts ~ Block + Block:BvsA + Block:CvsI + Block:BvsA:CvsI

Parametric coefficients:
                 Estimate Std. Error z value Pr(>|z|)
(Intercept)       0.97327    0.99534   0.978   0.3282
Block2           -3.63268    1.96579  -1.848   0.0646 .
Block1:BvsA      -0.04241    0.67177  -0.063   0.9497
Block2:BvsA       2.02107    0.97068   2.082   0.0373 *
Block1:CvsI      -0.28401    0.64521  -0.440   0.6598
Block2:CvsI       1.01199    1.06658   0.949   0.3427
Block1:BvsA:CvsI  0.25394    0.43270   0.587   0.5573
Block2:BvsA:CvsI -0.59977    0.62025  -0.967   0.3336
---
Signif. codes:  0  ***  0.001  **  0.01  *  0.05  .  0.1     1


R-sq.(adj) =  0.485   Deviance explained = 39.5%
-REML = 86.933  Scale est. = 1         n = 35
>
>  c(gam.baci.03$deviance,gam.baci.03$df.residual,qchisq(p=0.05,  df=gam.baci.03$df.residual,
ncp = 0,
+                    lower.tail = FALSE, log.p = FALSE))
[1] 34.41002 27.00000 40.11327
>
>
> vec <- matrix(data=rep(1,2), nrow=1, ncol=2)
>
> Interact_pars <- sum(gam.baci.03$coefficients[7:8])/2
> Interact_pars_SE <- (vec %*% (vcov(gam.baci.03)[7:8,7:8])) %*% t(vec)
> phi <- 1
> Interact_pars_SE <- (((phi)*Interact_pars_SE)^0.5)/2
```

```
> gam.baci.03$family$getTheta(TRUE)
[1] 11.57873
> phi <- gam.baci.03$deviance/gam.baci.03$df.residual
>
> CritH0 <- qnorm(p=0.05, mean = 0, sd = Interact_pars_SE, lower.tail = TRUE, log.p = FALSE)
> # calcuate Prob(Type II error) for a true reduction of
> PrTypeII <- pnorm(q=CritH0, mean =Interact_50pc, sd = Interact_pars_SE, lower.tail = FALSE,
log.p = FALSE)
> Power <- 1-PrTypeII
> Interact_R <- 100*(1-exp(Interact_pars))
> t_stat <- Interact_pars/Interact_pars_SE
> print(c(Interact_pars,Interact_pars_SE,t_stat,Interact_R,CritH0,PrTypeII,Power,phi))
[1] -0.1729183  0.3781323 -0.4572959 15.8793700 -0.6219722  0.4253491  0.5746509
[8]  1.2744451
>
>
```
